# Functional diversity of TMPRSS6 isoforms and variants expressed in hepatocellular carcinoma cell lines

**DOI:** 10.1038/s41598-018-30618-z

**Published:** 2018-08-22

**Authors:** Sébastien P. Dion, François Béliveau, Louis-Philippe Morency, Antoine Désilets, Rafaël Najmanovich, Richard Leduc

**Affiliations:** 1 Department of Pharmacology-Physiology, Faculty of Medicine and Health Sciences, Université de Sherbrooke, Sherbrooke, Québec Canada; 2 Institut de Pharmacologie de Sherbrooke, Faculty of Medicine and Health Sciences, Université de Sherbrooke, Sherbrooke, Québec Canada; 3 Department of Biochemistry, Faculty of Medicine and Health Sciences, Université de Sherbrooke, Sherbrooke, Québec Canada; 4 Department of Pharmacology and Physiology, Université de Montréal, Montréal, Québec Canada; 5 PROTEO – The Quebec Network for Research on Protein Function, Engineering and Applications, Québec, Canada

## Abstract

TMPRSS6, also known as matriptase-2, is a type II transmembrane serine protease that plays a major role in iron homeostasis by acting as a negative regulator of hepcidin production through cleavage of the BMP co-receptor haemojuvelin. Iron-refractory iron deficiency anaemia (IRIDA), an iron metabolism disorder, is associated with mutations in the *TMPRSS6* gene. By analysing RNA-seq data encoding TMPRSS6 isoforms and other proteins involved in hepcidin production, we uncovered significant differences in expression levels between hepatocellular carcinoma (HCC) cell lines and normal human liver samples. Most notably, *TMPRSS6* and *HAMP* expression was found to be much lower in HepG2 and Huh7 cells when compared to human liver samples. Furthermore, we characterized the common *TMPRSS6* polymorphism V736A identified in Hep3B cells, the V795I mutation found in HepG2 cells, also associated with IRIDA, and the G603R substitution recently detected in two IRIDA patients. While variant V736A is as active as wild-type TMPRSS6, mutants V795I and G603R displayed significantly reduced proteolytic activity. Our results provide important information about commonly used liver cell models and shed light on the impact of two TMPRSS6 mutations associated with IRIDA.

## Introduction

The human type II transmembrane serine proteases (TTSPs) are a family of proteolytic enzymes expressed on the surface of numerous cell types. One member of this family, TMPRSS6, also known as matriptase-2, is mainly expressed in the liver^1^. This protein is known to be an important player in iron homeostasis due to its negative regulatory effect on hepcidin production^2^. Hepcidin, encoded by the *HAMP* gene, is a circulatory hormone that controls blood iron levels by binding to and internalizing ferroportin, which is expressed on the surface of hepatocytes, macrophages and enterocytes, thus trapping iron intracellularly^3^. TMPRSS6 acts upstream of the hepcidin production signalling pathway by cleaving haemojuvelin (HJV) at the cell surface. HJV is a bone morphogenic protein (BMP) coreceptor encoded by the *HFE2* gene that leads to downstream regulation of the BMP/SMAD signalling pathway. The consequence of HJV cleavage diminishes signalling and ultimately reduces *HAMP* transcription^4,5^. Other important players in iron regulation include transferrin receptor 2 (*TFR2*) and human haemochromatosis protein (*HFE*), which intervene in the BMP/SMAD signalling pathway, leading to hepcidin production in response to holo-transferrin iron binding^6–8^, and transferrin receptor 1 (*TFRC*), involved in transferrin-bound iron uptake^9^.

Following cloning of *TMPRSS6*^1^, several *TMPRSS6* gene mutations have been found to be associated with iron-refractory iron deficiency anaemia (IRIDA, OMIM #206200), a rare type of anaemia characterized by a lack of response to oral iron therapy but with partial response to parenteral iron administration^10–14^. IRIDA is an autosomal hereditary recessive disease clinically characterized by hypochromic, microcytic anaemia and low saturation levels of serum iron and transferrin^15^.

Because of its physiological role in reducing hepcidin levels, it stands to reason that TMPRSS6 has become an attractive therapeutic target for diseases characterized by iron overload, such as hereditary haemochromatosis (OMIM #235200) and beta-thalassemia (OMIM #613985)^16–18^. Therefore, to further understand the role of TMPRSS6 in iron homeostasis, cellular models including primary hepatocytes and liver-derived hepatocyte cell lines, such as hepatocellular carcinoma (HCC) cells (Hep3B, HepG2, Huh7), have been widely used because they possess both the protein and signalling machinery controlling hepcidin expression^3,19–24^.

Herein, we describe important functional differences and variations in expression levels of *TMPRSS6*, its isoforms and other iron related genes in HCC cell lines when compared to liver samples. Moreover, we have identified six *TMPRSS6* single nucleotide polymorphisms (SNPs) in Hep3B and HepG2 cell lines. Using heterologous expression, we have characterized some properties of TMPRSS6 variant V736A and mutant V795I identified in Hep3B and HepG2 cell lines, respectively, and an uncharacterized mutant (G603R), found in two patients suffering from IRIDA^12,13^, thus providing insight into the molecular basis of IRIDA.

## Materials and Methods

### Cells, Antibodies, and Reagents

HEK293 cells were purchased from American Type Culture Collection (Manassas, VA). These cells were cultured in high glucose Dulbecco’s Modified Eagle’s Medium (DMEM) with 10% foetal bovine serum, 2 mM L-glutamine, 100 IU/ml penicillin and 100 µg/ml streptomycin. Serum-free media HCELL-100 was purchased from WISENT (St-Bruno, Canada). Poly-L-lysine coated coverslips were purchased from Corning (Bedford, MA). Anti-V5, Anti-V5 HRP and Anti-V5 FITC-linked monoclonal antibodies were purchased from Invitrogen (Waltham, MA). HRP-linked Anti-GAPDH rabbit monoclonal antibody was purchased from Cell Signaling Technology (Danvers, MA). Goat polyclonal anti-Hemojuvelin antibody and t-butoxycarbonyl-Gln-Ala-Arg-7-amino-4-methylcoumarin (Boc-QAR-AMC) were purchased from R&D Systems (Minneapolis, MN). Lipofectamine 3000 was purchased from Invitrogen (Carlsbad, CA). Centrifugal filters were purchased from Merck Millipore (Cork, Ireland). Lysis buffer (1% Triton, 50 mM Tris, 150 mM NaCl, 5 mM EDTA) was supplemented with protease inhibitor from Roche (Mannheim, Germany). Protein A/G PLUS-agarose beads were purchased from Santa-Cruz Biotechnology (Dallas, TX).

### RNA-sequencing (RNA-seq) data analysis

Expression of *TMPRSS6* transcripts and iron-related genes in human tissue samples (RPKM; reads per kilobase of exon per million fragments mapped) were obtained from the Genotype-Tissue Expression (GTEx) project (release V6p)^25^. All available GTEx liver data sets were analysed, and their sample identification numbers (id) are listed in Table S1. Expression in Hep3B, HepG2 and Huh7 cell lines was obtained by analysing publicly accessible RNA-seq datasets from at least three different studies without any specific selection criteria. Sequences from each cell line were retrieved from the European Nucleotide Archive. The accession numbers used are listed in Table S2. The obtained paired-end reads from RNA-seq datasets were aligned to the human reference genome GRCh37/hg19 using HISAT2 v2.03^26^. Genes and transcript RPKM expression values were calculated with Cufflinks v2.2.1.0^27^ using the annotated transcriptome from ENSEMBL (ftp://ftp.ensembl.org/pub/release-75/gtf/homo_sapiens) as a reference. TMPRSS6 isoforms numbering (1 to 4) is based according UniProt nomenclature^28^. TMPRSS6 variants can be referenced as follows: P33P (NG_012856.2:g.11218 G > A, rs11704654), Y418Y (NG_012856.2:g.39314 C > T, rs881144), C459C (NG_012856.2:g.30423 T > C, rs2543520), I430T (NG_012856.2:g.30335 T > C, rs2543519), V736A (NG_012856.2:g.47668 T > C, rs855791), V795I (NG_012856.2:g.48431 G > A, rs139105452), G603R (NG_012856.2:g.44019 G > C, rs769083817).

### Plasmid construction

cDNA encoding TMPRSS6-2 WT, TMPRSS6-1 S762A and HJV were obtained and cloned as previously described^23,24^. TMPRSS6-2 constructs V736A, V795I, G603R and S762A were obtained using the QuikChange site-directed mutagenesis kit (Agilent Technologies, Santa Clara, CA). Primers used are listed in Table S3. Numbering refers to TMPRSS6 isoform 1 sequence (residues 1 to 811).

### Immunofluorescence

HEK293 cells were seeded on poly-L-lysine coated coverslips and transfected with 2 µg of TMPRSS6-2 DNA constructs using Lipofectamine 3000 in 6-well plates. Twenty-four hours later, cell surface TMPRSS6 was labelled for 1 hour at 4 °C. Cells were washed and prepared directly as previously described^29^ using ProLong Diamond Antifade Mountant with DAPI (Invitrogen, Eugene, OR) or incubated at 37 °C for 15 or 30 min in DMEM 10% FBS prior to preparation. Cells were examined using a Plan Apo 60x oil immersion objective NA 1.42 on an inverted spectral scanning confocal microscope FV1000 (Olympus, Tokyo, Japan). Laser excitation was performed at 405 nm (50 mW Violet diode laser) and 488 nm (40 mW Blue Argon Laser). Images were pseudocoloured according to their original fluorochrome and merged using FluoView software (Olympus, Tokyo, Japan) as previously described^24^.

### Expression, shedding and proteolytic activity of TMPRSS6-2 variants

HEK293 cells were transfected with 2 µg of TMPRSS6-2 DNA constructs using Lipofectamine 3000 in 6-well plates. Twenty-four hours later, cell media was replaced with HCELL-100 media for 24 hours. Cell media was collected, and 1 mL was concentrated before cells were lysed. Samples (30 µg of cell lysate and 30 µL of concentrated media) were loaded on 12% SDS-PAGE and analysed by immunoblotting using anti-V5 and anti-GAPDH antibodies as previously described^24^. Proteolytic activity was measured using unconcentrated cell media to monitor Boc-QAR-AMC cleavage as previously described using a FLx800 TBE microplate reader (Bio-Tek Instruments, Winooski, VT)^23,24,30^.

### Haemojuvelin processing by TMPRSS6 variants

HEK293 cells were co-transfected with 1 µg of TMPRSS6-2 constructs and 1 µg of haemojuvelin transcript A. Cells were grown in HCELL-100 media for 24 hours as previously described^24^. Cell media (1 mL) was then collected and concentrated before lysing the cells. Cell lysate (30 µg) and concentrated media (30 µL) were loaded on SDS-polyacrylamide gels and analysed with immunoblotting using anti-HJV, anti-V5 and anti-GAPDH antibodies as previously described^24^.

### Interaction between TMPRSS6 variants and HJV

HEK293 cells were co-transfected with 1 µg of TMPRSS6-2 constructs and 1 µg of haemojuvelin transcript A. At 24 hours post-transfection, cells were washed and harvested on ice in 300 µL of lysis buffer. Protein samples (400 µg) were immunoprecipitated in 500 µL volume with an anti-V5 antibody (2.4 µg/mL) and Protein A/G PLUS-agarose beads for 24 hours at 4 °C. Immunoprecipitated proteins were loaded on SDS-polyacrylamide gels and analysed by immunoblotting using anti-HJV and anti-V5 antibodies as previously described^24^.

### Statistical analysis

Statistical analyses were conducted using GraphPad Prism version 7.0c (GraphPad Software, La Jolla, CA). For proteolytic activity, outliers were removed using the ROUT method (Q = 1%). Normality was assessed using the D’Agostino-Pearson omnibus normality test before using the nonparametric Kruskal-Wallis test. Statistical significance was assumed at *P* < 0.05.

## Results

### Hepatocellular carcinoma cell lines as models of TMPRSS6-iron regulation

Previously, our group has identified and characterized four distinct human TMPRSS6 isoforms by analysing publicly available RNA-seq data^24^. *TMPRSS6* isoform 1 (*TMPRSS6-1*), known as the *TMPRSS6* canonical isoform according to UniProt^28^, was not detected in human liver. *TMPRSS6-2*, which differs from *TMPRSS6-1* by the absence of 9 amino acids in the N-terminal cytoplasmic tail of the protein, is the most expressed isoform in the liver, pituitary and testis^24^. Data suggest that *TMPRSS6-3*, an isoform without a catalytic domain and hence proteolytically inactive, is expressed at negligible levels in human liver^24^. Finally, *TMPRSS6-4*, which contains an insertion in its catalytic domain and is proteolytically inactive, is a relatively abundant isoform detected in human liver^24^.

**Figure 1:**
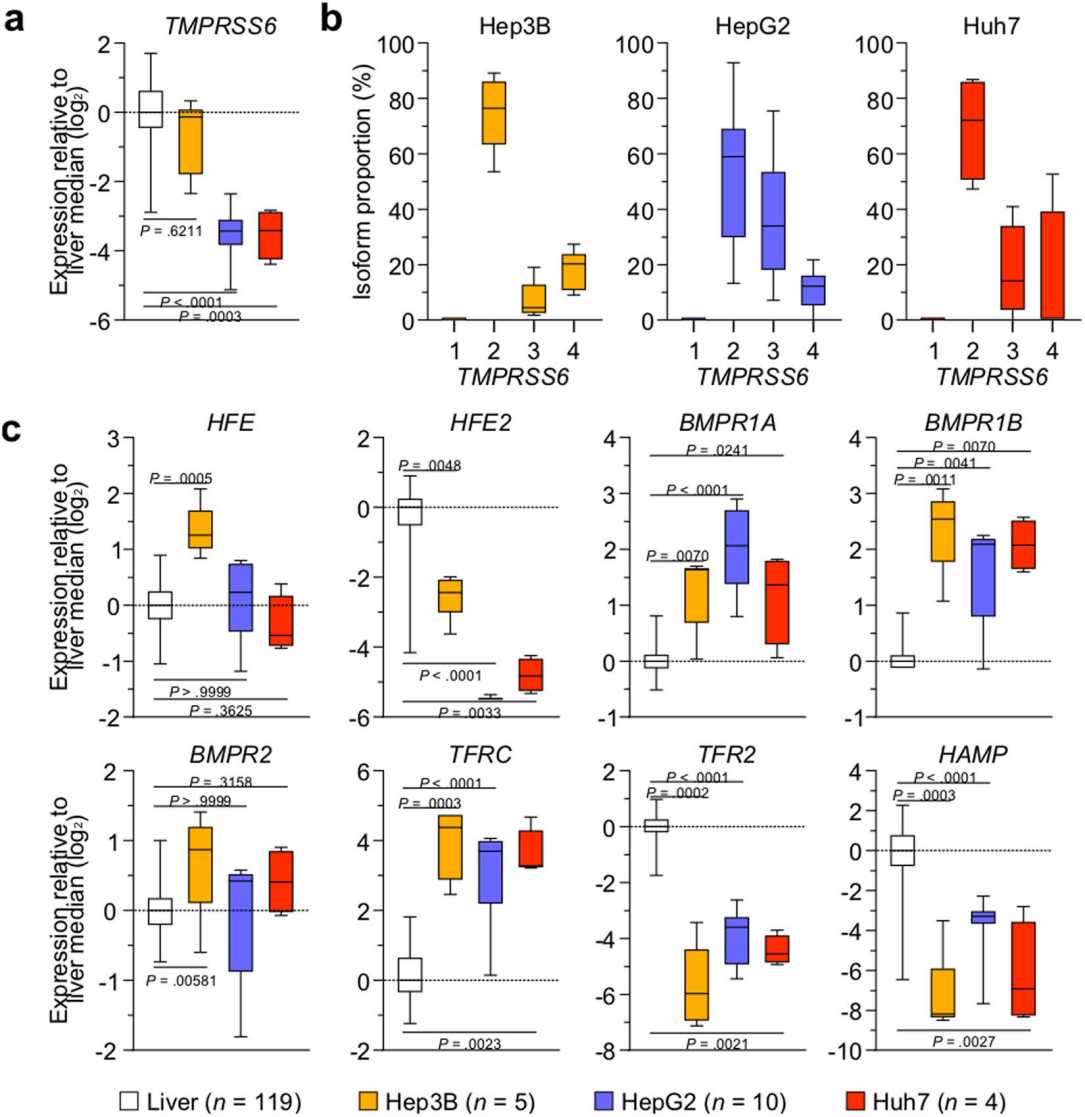
*TMPRSS6* and iron regulation associated gene expression in human liver and hepatocellular carcinoma cell lines. (**a**) Relative *TMPRSS6* expression levels in hepatocellular carcinoma (HCC) cell lines. Median expression results are presented as log_2_ of RPKM + 1 and are compared to liver samples. (**b**) *TMPRSS6* isoform proportional expression in HCC cell lines. The results are presented as percentages of isoform expression. (**c**) Hepcidin production-related genes relative expression. The results are presented as log_2_ RPKM + 1 and are compared to liver samples. All results were obtained using RNA-seq data analysis. Box-and-whisker plots display quartiles and range. Statistical significance was assessed with the Kruskal-Wallis multiple comparison test.

Because HCC cell lines are frequently used to study the signalling pathway leading to hepcidin production, we analysed RNA-seq datasets originating from at least three separate studies for each commonly used HCC cell line: Hep3B, HepG2 and Huh7. First, we examined global *TMPRSS6* expression levels (Fig. 1a) and the proportion of differently expressed *TMPRSS6* isoforms (Fig. 1b). When compared to normal human liver samples, global *TMPRSS6* expression was similar in Hep3B cells but significantly lower (90%) in HepG2 and Huh7 cells (Fig. 1a).

The proportion of TMPRSS6 isoforms also varied between HCC cell lines. In all three lines tested, *TMPRSS6-1* was not expressed, and *TMPRSS6-2* was the most abundant transcript detected (Fig. 1b). Furthermore, inactive *TMPRSS6-4* is expressed substantially in HCC cell lines (Fig. 1b) at similar levels to those found in the liver^24^. Interestingly and in contrast to the liver^24^, all three cell lines Hep3B (5%), HepG2 (34%), Huh7 (14%) expressed the inactive *TMPRSS6-3* isoform at substantial levels (Fig. 1b).

We next examined if the expression levels of other genes associated with iron regulation, such as haemochromatosis (*HFEs*), bone morphogenetic protein receptors (*BMPRs*), transferrin receptor (*TFRs*) and hepcidin (*HAMP*), varied between human liver and HCC cell lines (Fig. 1c). *BMPR2* and *HFE* levels varied little, while levels of *HFE2* (coding for HJV), *TFR2* and *HAMP* were significantly lower in all three cell lines compared to liver samples. In contrast, *TFRC*, *BMPR1A* and *BMPR1B* transcripts levels were higher in all HCC cell lines (Fig. 1c). Thus, HCC cell lines diverge from liver samples with regards to *TMPRSS6* expression and more generally to important regulators of the HJV-BMP-hepcidin signalling axis.

**Figure 2:**
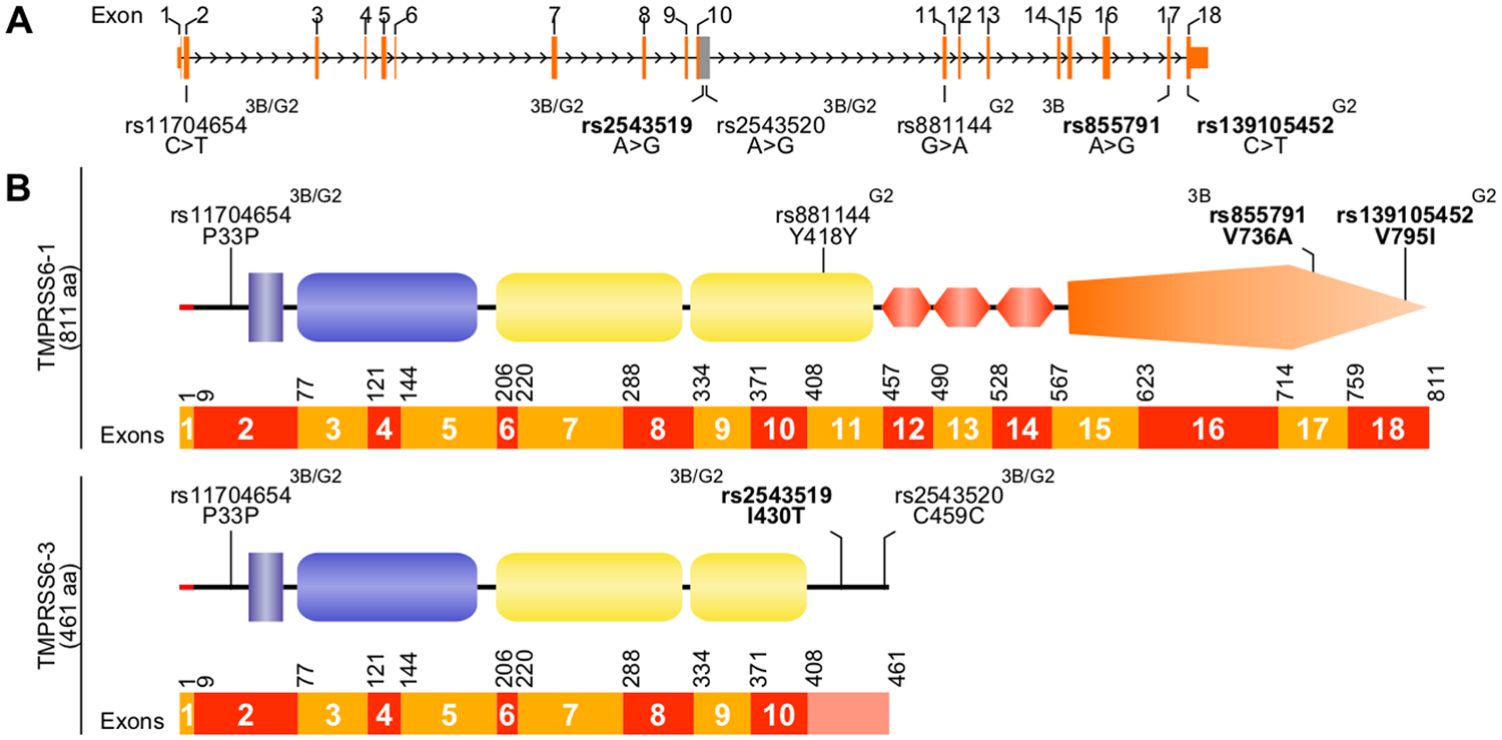
TMPRSS6 variants detected in Hep3B and HepG2 cell lines. (**A**) Single nucleotide polymorphisms (SNPs) detected by RNA-seq data analysis are displayed with their SNP reference ID number (rs) on *TMPRSS6* gene representation. Coding regions are displayed taller than non-coding regions. (**B**) SNPs leading to silent mutations or missense variations (regular font) or mutations (bold) are presented on TMPRSS6-1 and 3 scaffolds. The nucleotide numbering was conserved using TMPRSS6-1 (residues 1 to 811) as a reference. On this figure, Hep3B is abbreviated 3B and HepG2 is abbreviated G2. TMPRSS6 schematic representation is adapted from our previous publication^24^ licenced under CC BY 3.0.

While assessing *TMPRSS6* isoform expression in HCC cell lines, we also identified six *TMPRSS6* homozygous single nucleotide polymorphisms (SNPs) in Hep3B and HepG2 cells (Fig. 2a). Three SNPs were identified in both cell lines. Of these, one led to a silent mutation (P33P, rs11704654) found in all isoforms, and the two others were a missense mutation (I430T, rs2543519) and silent mutation (C459C, rs2543520) that affected *TMPRSS6* isoform 3 specifically (Fig. 2b). Interestingly, mutation I430T was previously identified in IRIDA patients^31^ and associated with breast cancer risk and poorer prognosis^32^, but it was never specified that this mutation is located in a region that is exclusive to the inactive isoform 3 (elongated exon 10, Fig. 2b).

A fourth SNP encoding the V736A polymorphism (rs855791),was detected in Hep3B cells (Fig. 2b). This variant is mainly found in African populations^33^ and is associated with lower haemoglobin levels^34,35^. Moreover, it has been found to be detrimental in patients suffering from non-transfusion-dependent thalassemias^36^ and identified in patients with iron deficiency anaemia (IDA), a milder form of IRIDA partially responsive to iron treatment, suggesting the polymorphism has a protective effect^11,37^. On the other hand, valine at position 736 (V736) has been identified as beneficial in non-alcoholic fatty liver disease (OMIM %613282)^38^. Another mutation found at this position (V736D) was also previously associated with IRIDA^39^.

The last two SNPs were detected in HepG2 cells. One led to a silent mutation (Y418Y, rs881144) while the other to a missense mutation (V795I, rs139105452), which has already been associated with patients suffering from IRIDA^11,40,41^.

### Functionalities of TMPRSS6 variants

TMPRSS6 mutations have been frequently associated with enzyme dysfunction^42,43^. Therefore, we investigated the effect of the V736A variant (Hep3B) and the missense mutation V795I (HepG2) on the protease’s properties. We compared these mutants to TMPRSS6 isoform 2 along with the uncharacterized G603R mutation (rs769083817) found in two patients suffering from IRIDA^12,13^. Notably, two other missense mutations are annotated at position 603 according to ExAC Browser^44^ but are not associated with IRIDA. The V736A variant and the V795I and G603R mutants were cloned in the TMPRSS6-2/V5 backbone because *TMPRSS6-2* is the main transcript expressed in HCC cells lines (Fig. 1b) and human tissues^24^.

**Figure 3:**
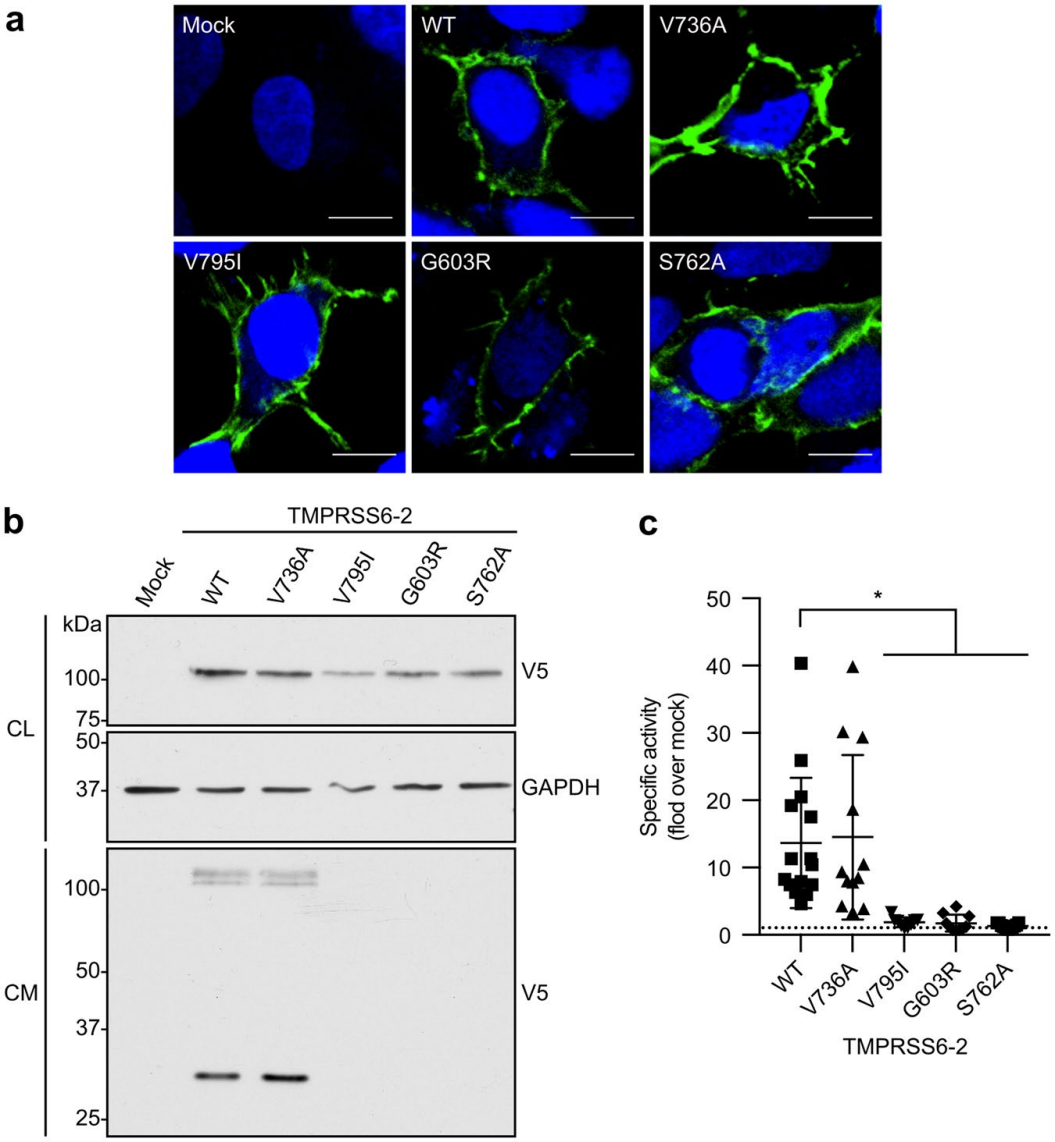
TMPRSS6 variants’ functionalities in HEK293 transfected cells. (**a**) Cell surface expression of TMPRSS6-2/V5 variants. Cells were grown on poly-L-lysine coverslips and transfected with TMPRSS6-2/V5 variants. Cells were surface-labelled with anti-V5 FITC antibody prior to processing for confocal microscopy analysis. Anti-V5 FITC immunofluorescence is displayed in green, and the DAPI stained nucleus is shown in blue (scale bars: 10 µm, n ≥ 3). (**b**) Cells were transfected with TMPRSS6-2/V5 variants, and expression was detected by immunoblotting with an anti-V5 antibody. Equal amounts of cell lysate (CL) and concentrated cell medium (CM) were loaded on 12% SDS-polyacrylamide gels. Cell lysate GAPDH was blotted as a loading control (n ≥ 3). Full-length blots are presented in Figure S2. (**c**) Proteolytic activity was measured in the cell medium of cells transfected with TMPRSS6-2/V5 variants. The fluorescence released by the cleavage of Boc-QAR-AMC (200 µM) was monitored. The results are presented as specific activity (fold over mock, fluorescence units/µL/µg of total proteins), are baseline corrected and are shown as scatter pot ± SD (n ≥ 10). Statistical significance was assessed by the Kruskal-Wallis multiple comparison test. *P* values < 0.05 were considered statistically significant (*).

Because specific mutations are known to affect the protein’s subcellular localization^43^, we initially verified the ability of TMPRSS6 variants to translocate to the cell surface as previously described for TMPRSS6 isoforms using heterologous expression in HEK293 transfected cells^24^. Using confocal microscopy, we confirmed that variants V736A, V795I, G603R and the catalytically inactive mutant S762A (control) reached the cell surface (Fig. 3a).

Since all variants translocated to the cell surface, we next verified their capacity to undergo auto-activation. At comparable zymogen expression levels (as detected by the >100 kDa band in the cell lysate), the V736A variant showed similar shedding levels to TMPRSS6-2 WT, as seen by the ~30 kDa band (Fig. 3b) detected in the cell media. Expression of the V795I, G603R and catalytically inactive S762A mutations led to a significant reduction in cell-surface shedding (Fig. 3b), suggesting that the proteolytic activity of the mutant proteins was altered. Consequently, the proteolytic activity in the extracellular medium of cells transfected with the different mutants was measured and compared to TMPRSS6-2 WT (Fig. 3c). Similar to the shedding results, we detected no differences in proteolytic activity between variant V736A and TMPRSS6-2 WT, while mutants V795I, G603R and S762A demonstrated significantly lower activity levels in media (Fig. 3c).

**Figure 4:**
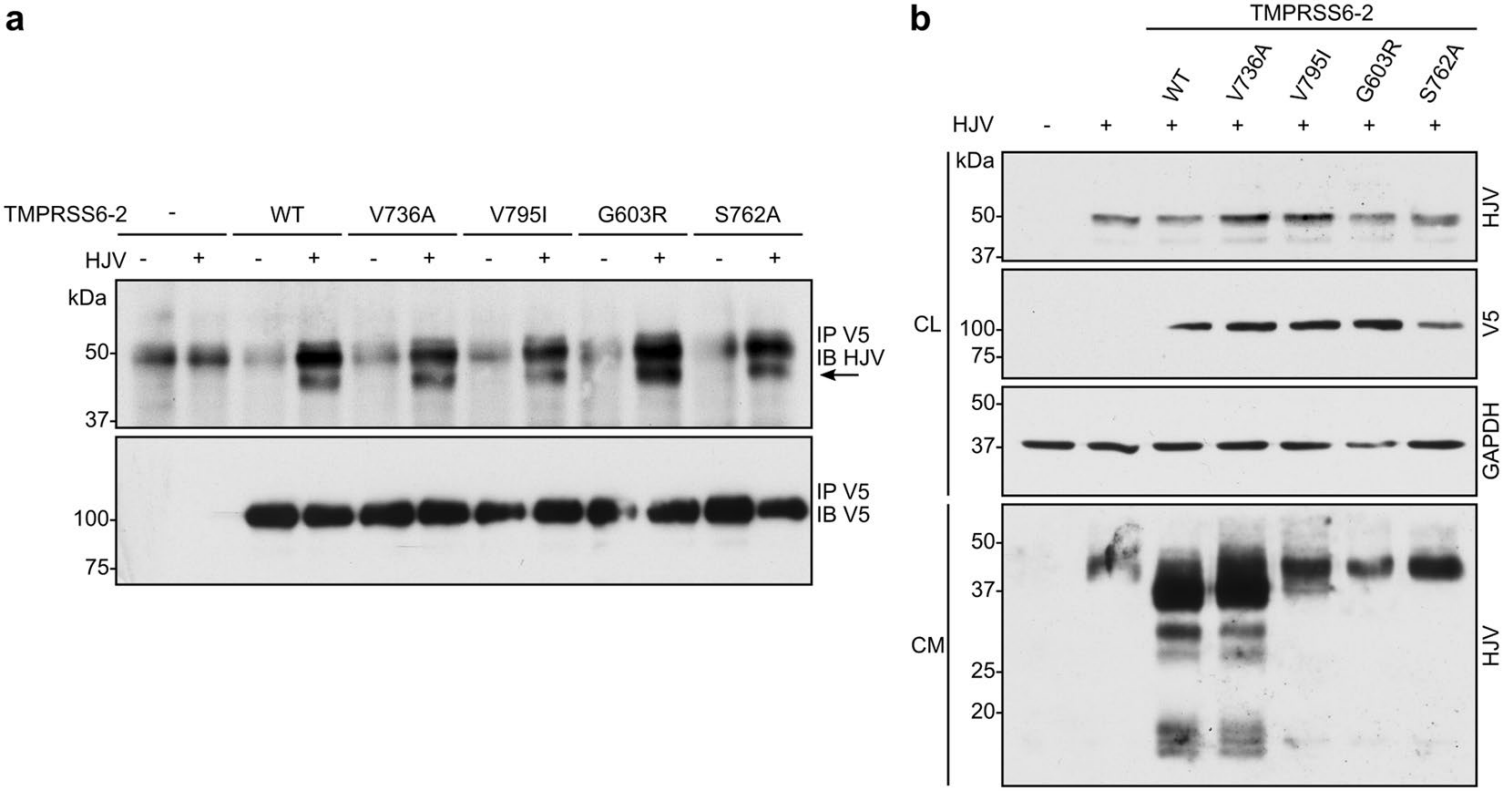
TMPRSS6 variants’ interaction with HJV. (**a**) HEK293 cells were co-transfected with TMPRSS6-2/V5 variants and HJV. Immunoprecipitation was performed in cell lysates using an anti-V5 antibody, and immunoblotting was performed using anti-HJV or anti-V5 antibodies (n ≥ 3). (**b**) HEK293 cells were co-transfected with TMPRSS6-2/V5 variants and HJV. HJV cleavage in cell media was detected by immunoblotting with anti-HJV antibody. Equal amounts of cell lysate (CL) and concentrated extracellular medium (CM) were loaded on 12% SDS-polyacrylamide gels. Cell lysate GAPDH was blotted as a loading control (n ≥ 3). Full-length blots are presented in Figure S2.

We next verified if all TMPRSS6 variants interacted with HJV, a TMPRSS6 substrate^4^. Co-immunoprecipitation showed that all TMPRSS6 variants interacted with HJV as indicated by the ~50 kDa band present only in co-transfected conditions (Fig. 4a). We then determined the mutants’ capacity to cleave HJV. While variant V736A cleaved HJV similarly to TMPRSS6-2 WT, as seen by the appearance of immunoreactive bands below 37 kDa in the cell media, mutants V795I, G603R and S762A showed little or no cleavage of HJV (Fig. 4b, *lower panel*), which is the result of their low proteolytic activity.

## Discussion

Hepatocellular carcinoma Hep3B, HepG2 and Huh7 cell lines have frequently been used to study signalling pathways related to iron homeostasis. TMPRSS6 is at the apex of BMP/SMAD signalling involved in hepcidin production and is an important player involved in iron homeostasis. To better understand the role of TMPRSS6 in these cell types, our study addresses the expression levels of *TMPRSS6* transcripts, the nature of its isoforms and the global expression levels of several other hepcidin-iron-related genes in these cell lines in comparison to human liver samples.

By performing transcriptomic analysis of publicly available RNA-sequencing (RNA-seq) data, we show equivalent global expression levels of *TMPRSS6*, all isoforms taken together, in the Hep3B cell line when compared to the liver. However, HepG2 and Huh7 cell lines display significantly lower expression levels of *TMPRSS6* transcripts.

Our data also reveal that an often-studied TMPRSS6 isoform in the context of hepatocyte-dependent iron regulation, *TMPRSS6-1*^4,14,23,42,45,46^, is not expressed in HCC cell lines. Interestingly, *TMPRSS6-1* expression levels are also undetectable in human liver samples, as previously demonstrated^24^. *TMPRSS6* isoform expression analysis in HCC cell lines also emphasizes the potential role of functionally altered TMPRSS6 isoform 3 in cancer, as there are discrepancies between HCC cells lines and healthy human liver samples. The expression and role of this isoform in cancer progression is of particular interest because of its potential role to act as a dominant-negative protein^24^. It is important to note that in this study, we report differences in TMPRSS6 transcript both in type and abundance based on mRNA quantifications. To ascertain that these differences translate to changes at the protein level, extensive mass spectrometry and antibody-based assays will need to be developed to distinguish between the closely related isoforms.

*HAMP* transcript levels are noticeably lower in all three HCC cell lines when compared to human liver samples. *TMPRSS6* and *HAMP* transcript expression patterns indicate many potential differences in the BMP/SMAD signalling pathway, leading to hepcidin production between cell lines and tissues. This is validated by the fact that expression levels of transcripts involved in the TMPRSS6/Hepcidin axis differ between HCC cell lines and human liver samples. Of note, transcript levels of *HFE2*, which encodes HJV, are significantly lower in all three HCC cell lines studied compared to human liver samples.

Differences in transcript expression levels should be considered in parallel with mutations detected in these cancerous cell lines. For instance, even though *HFE* transcripts levels vary less than others, it is worth mentioning that a mutation of this gene has already been identified in Huh7 cell lines, thus positioning this HCC cell line as a model of human haemochromatosis^22^. Analysing RNA-seq data, we identified six *TMPRSS6* SNPs in Hep3B and HepG2 cell lines, three of which lead to known amino acid substitutions of the TMPRSS6 protein. Using HEK293 cells as a cellular model, we show that the common V736A polymorphism variant identified in Hep3B cells does not affect TMPRSS6 cell surface expression or its catalytic activity when compared to TMPRSS6 WT. Because V736A has been associated with higher susceptibility to hepatic iron accumulation in thalassemia patients^36^ and lower hepcidin levels in normal individuals^35^, and because of the predicted entropy gain assessed by our structural bioinformatics analysis^47,48^, we expected higher catalytic activity of the V736A variant. It is possible that the gain of function is subtle and could not be measured in our cellular enzymatic assay.

We also demonstrated that mutations V795I, detected in HepG2, and G603R, which are both associated with IRIDA^11–13,40,41^, affect the enzyme’s capacity to undergo autocatalysis. In our assays, these mutants maintained their ability to interact with HJV but were unable to cleave it, strongly suggesting a loss of TMPRSS6 proteolytic activity. When overexpressed in HEK293 cells, mutations V795I and G603R displayed severe loss of function, suggesting their role in hepcidin up-regulation and with the IRIDA phenotype. This loss of function is supported by predicted loss of vibrational entropy of the enzyme’s catalytic domain combined with an unfavourable enthalpy penalty as assessed using structural thermodynamic analysis (Fig. S1)^48^. However, we cannot rule out that V795I and G603R variants could present a certain level of proteolytic activity under different conditions. The effect on the activity of these variants could also result in impaired cleavage of other TMPRSS6 substrates involved in hepcidin regulation. Indeed, using mouse homologues, it has recently been shown that apart from HJV, TMPRSS6 could process other components of the hepcidin induction pathway including BMP receptors, Hfe and Tfr2^49^. Intriguingly, reports on the V795I mutant^40^ showed that it still reduces HAMP-driven luciferase activity. Other reports have also shown that proteolytically inactive TMPRSS6 mutants still reduce HAMP-driven luciferase activity *in vitro*^4,40,50^. In these overexpression assays, the negative effect of inactive TMPRSS6 mutants in HAMP-driven luciferase assays has been proposed to be artefactual, and one report suggested that catalytic activity measurement should be prioritized^50^. However, others claim that the HAMP-driven luciferase assay may be more sensitive than proteolytic activity measurement if performed under controlled conditions^51^. Further studies are needed to better understand the V795I and G603R dysfunctionalities and thus the molecular basis of particular cases of IRIDA associated with these mutations.

We believe the results presented herein provide important information for future studies using primary hepatocytes and HCC cells to examine various mechanistic aspects related to iron metabolism. Importantly, we have revealed the presence of transcripts coding for functionally altered TMPRSS6 isoforms and loss of function mutations in the commonly used Hep3B and HepG2 cell lines, which may address potential issues in iron regulation studies. Hence, we emphasize the importance of ascertaining gene expression and protein functionality when using cellular models to study TMPRSS6 function. In fact, the development and use of human cellular models more comparable to normal hepatocytes (primary cells or iPSC-derived) could be considered as an added complement to validate data obtained in HCC cell lines with regards to complex processes such as iron regulation.

### Data availability

All data generated or analysed during this study are included in this published article (and its Supplementary Information files).

## Electronic supplementary material

Supplementary Information
