# Supplementary material for: Functional diversity of TMPRSS6 isoforms and variants expressed in hepatocellular carcinoma cell lines
Source: Sci Rep. 2018 Aug 22;8:12562. doi: 10.1038/s41598-018-30618-z (PMC6105633; doi:10.1038/s41598-018-30618-z)
Supplement: Supplementary file 1 — Supplementary Information [file 41598_2018_30618_MOESM1_ESM.pdf]

Functional diversity of TMPRSS6 isoforms and variants expressed in hepatocellular carcinoma cell lines

Running title: TMPRSS6 isoforms and variants in liver cell lines

Sébastien P. Dion[1,2], François Béliveau[1,2], Louis-Philippe Morency[2,3,4,5], Antoine Désilets[1,2], Rafaël Najmanovich[4,5], Richard Leduc[1,2*].

[1] Department of Pharmacology-Physiology, Faculty of Medicine and Health Sciences, Université de Sherbrooke, Sherbrooke, Québec, Canada

[2] Institut de Pharmacologie de Sherbrooke, Faculty of Medicine and Health Sciences, Université de Sherbrooke, Sherbrooke, Québec, Canada

[3] Department of Biochemistry, Faculty of Medicine and Health Sciences, Université de Sherbrooke, Sherbrooke, Québec, Canada

[4] Department of Pharmacology and Physiology, Université de Montréal, Montréal, Québec, Canada

[5] PROTEO – The Quebec Network for Research on Protein Function, Engineering and Applications

Supplementary information

Material and Methods

Effects of mutations on protein stability and dynamics

The evaluation of TMPRSS6 amino acid substitutions on thermodynamic stability was performed using a combination of two methods based on the homology model of the TMPRSS6 WT catalytic domain (isoforms 1 and 2) that was previously described.[1] ENCoM[2] was used to calculate the difference in vibrational entropy between TMPRSS6-1/2 WT, variants V736A and mutants V795I and G603R. FoldX[3] was used to calculate the difference in enthalpy between the variants and the WT catalytic domain. Using a linear combination of these two methods, we evaluated the thermodynamic properties of the resulting mutant structures as previously described.[2,4,5] Numbering refers to the TMPRSS6 isoform 1 catalytic domain sequence (residues 577 to 811).

Tables and Figures

Table S1: Liver samples datasets used from the Genotype-Tissue Expression (GTEx) Project

GTEx samples id for liver samples		
GTEX-T6MN-1226-SM-3NMA5	GTEX-14AS3-0126-SM-5Q5F4	GTEX-13FTW-1126-SM-5J2NV
GTEX-11NV4-1326-SM-5HL6V	GTEX-147JS-1126-SM-5RQIW	GTEX-12696-0826-SM-5EGGE
GTEX-131XH-0626-SM-5LZWH	GTEX-11DXZ-0126-SM-5EGGY	GTEX-P78B-1326-SM-3P611
GTEX-RN64-1826-SM-48FDV	GTEX-139TS-1426-SM-5IFJD	GTEX-ZAB4-0826-SM-5LU9D
GTEX-13VXU-0926-SM-5IFFH	GTEX-UPIC-0926-SM-4IHLV	GTEX-12WSG-0626-SM-5FQTQ
GTEX-11NUK-1226-SM-5P9GM	GTEX-13OW6-2626-SM-5IFF2	GTEX-RM2N-1926-SM-48FCU
GTEX-13FTZ-0726-SM-5IFFY	GTEX-147F4-1426-SM-5LUA8	GTEX-13113-1326-SM-5GCOI
GTEX-13SLX-1226-SM-5S2Q6	GTEX-12ZZZ-1326-SM-59HKW	GTEX-1497J-0726-SM-5Q5D1
GTEX-ZYT6-0626-SM-5E45V	GTEX-RWSA-1426-SM-47JXA	GTEX-14E1K-0326-SM-5S2PE
GTEX-WZTO-0626-SM-4PQYY	GTEX-11GSP-0626-SM-5986T	GTEX-UPK5-1426-SM-4JBHH
GTEX-QEG4-1826-SM-4R1JN	GTEX-14753-1626-SM-5NQ9L	GTEX-1269C-0626-SM-5FQSS
GTEX-13NYB-1026-SM-5IFH3	GTEX-X4EO-1126-SM-4QARQ	GTEX-YFC4-1526-SM-5IFJS
GTEX-ZVT3-1626-SM-5GU66	GTEX-X4EP-1026-SM-4QAS5	GTEX-144GM-1326-SM-5LU5E
GTEX-11DXY-0526-SM-5EGGQ	GTEX-XOTO-0826-SM-4B65O	GTEX-UTHO-2426-SM-4JBHD
GTEX-Q2AG-1126-SM-48U1P	GTEX-ZF2S-3026-SM-4WWCH	GTEX-145MO-2326-SM-5NQ9K
GTEX-QESD-2026-SM-447BI	GTEX-12KS4-1326-SM-5LUB3	GTEX-13PVR-0126-SM-5S2PY
GTEX-XBEC-1526-SM-4AT68	GTEX-X3Y1-2726-SM-4PQZH	GTEX-14C38-1526-SM-5RQJ7
GTEX-SJXC-1226-SM-4DM78	GTEX-11ZVC-0726-SM-5FQT9	GTEX-VUSG-0126-SM-4KL1X
GTEX-11ZUS-2526-SM-59872	GTEX-13NZB-0626-SM-5IFH6	GTEX-S33H-1626-SM-4AD68
GTEX-131YS-1626-SM-5HL6C	GTEX-TKQ2-1726-SM-4DXUP	GTEX-S4Z8-0526-SM-4AD4T
GTEX-145MF-0826-SM-5QGQA	GTEX-14A5I-1726-SM-5QGQ5	GTEX-QEL4-1226-SM-447A4
GTEX-YECK-1926-SM-4W21H	GTEX-R53T-0326-SM-48FEC	GTEX-139YR-0226-SM-5IFEM
GTEX-145LU-1326-SM-5LU9N	GTEX-132NY-0926-SM-5P9G3	GTEX-WQUQ-1926-SM-4OOSA
GTEX-1399T-0826-SM-5IFES	GTEX-RU72-1426-SM-46MUF	GTEX-ZPU1-0826-SM-57WG2
GTEX-12WSI-0226-SM-5GCNA	GTEX-13O3O-1826-SM-5IFGW	GTEX-X4XY-1626-SM-46MVN
GTEX-11OF3-0726-SM-5BC4Z	GTEX-ZAB5-0426-SM-5CVMI	GTEX-13N11-0926-SM-5IJG2
GTEX-13112-1426-SM-5EGH8	GTEX-ZVP2-0626-SM-51MSO	GTEX-ZYY3-0626-SM-5NQ6W
GTEX-PX3G-0826-SM-48TZS	GTEX-ZVT4-0626-SM-5E45T	GTEX-146FH-1526-SM-5NQBU
GTEX-RNOR-1426-SM-48FDJ	GTEX-QV44-0326-SM-4R1KD	GTEX-ZTPG-1426-SM-51MT3
GTEX-RTLS-1326-SM-46MUN	GTEX-13N2G-0926-SM-5IFGJ	GTEX-Y5LM-0426-SM-4VBRO
GTEX-13FLV-0326-SM-5N9DJ	GTEX-X261-1726-SM-4PQYT	GTEX-Z9EW-0426-SM-5CVM9
GTEX-1212Z-0226-SM-59HLF	GTEX-YB5E-0326-SM-5IFHU	GTEX-U3ZN-0226-SM-3DB8D
GTEX-12WSM-0726-SM-5GCOW	GTEX-11EQ9-0526-SM-5A5JZ	GTEX-12WSL-0226-SM-5CVMJ
GTEX-11WQC-0726-SM-5EQMR	GTEX-WFON-1726-SM-4LVMQ	GTEX-13NZ9-1326-SM-5MR3V
GTEX-11ZTS-1426-SM-5EQMM	GTEX-ZZPU-0426-SM-5GZYH	GTEX-131XE-0326-SM-5LZVO
GTEX-Y5V5-0926-SM-4VBPZ	GTEX-YEC4-0826-SM-5P9FV	GTEX-S32W-1926-SM-4AD63
GTEX-14DAQ-1726-SM-5S2R2	GTEX-REY6-1226-SM-48FDR	GTEX-QDVN-0826-SM-48TZ2
GTEX-1399R-1226-SM-5P9GF	GTEX-WYVS-1926-SM-4PQZ2	
GTEX-13QJC-0726-SM-5RQJK	GTEX-ZF29-2026-SM-4WWB7	
GTEX-139TU-0826-SM-5IJFG	GTEX-OOBJ-0826-SM-3NB2K	
GTEX-12WSD-1426-SM-5GCN9	GTEX-Q734-0326-SM-48U15	

Table S2. Accession numbers used for RNA-seq analysis of HCC cell lines.

Study	GEO Accession	BioSample	SRA	Run
**Hep3B**				
GSE70537	GSM1808716	SAMN03840429	SRX1081585	SRR2087590
GSE70537	GSM1808718	SAMN03840431	SRX1081587	SRR2087592
GSE49994	GSM1211428	SAMN02318973	SRX336427	SRR953772
		SAMN02580978	SRX434118	SRR1119486
GSE57666	GSM1386369	SAMN02777547	SRX542617	SRR1287012
**HepG2**				
GSE76173	GSM1975772	SAMN04351206	SRX1492027	SRR3033309
GSE30567	GSM758575	SAMN00634086	SRX1492027	SRR307926
GSE87958	GSM2343097	SAMN05896891	SRX2244585	SRR4422304
GSE88347	GSM2343829	SAMN05897111	SRX2244221	SRR4421932
GSE88654	GSM2344353	SAMN05897729	SRX2244753	SRR4422482
GSE87996	GSM2343161	SAMN05897066	SRX2244916	SRR4422653
GSE88362	GSM2343855	SAMN05897363	SRX2244247	SRR4421958
GSE78686	GSM2072617	SAMN04284443	SRX1603669	SRR3192697
GSE90322	GSM2400319	SAMN05733859	SRX2370637	SRR5048398
GSE86659	GSM2308416	SAMN05733829	SRX2156837	SRR4235539
**Huh7**				
		SAMN02377722	SRX365521	SRR1020608
GSE52530	GSM1269363	SAMN02412893	SRX379584	SRR1032892
		SAMEA3669191	ERX1217494	ERR1138633
		SAMEA3669193	ERX1217496	ERR1138634

Table S3. Primers and fragments used for cloning.

TMPRSS6 construct	5'- 3' Forward	5'- 3' Reverse
TMPRSS6-2 S762A (from TMPRSS6-1 S762A construct)	TTAAACTTAAGCTTGGTACCATGCCCGTGGCCGAGGCCCC	GGGGCCTCGGCCACGGGCATGGTACCAAGCTTAAGTTTAA
TMPRSS6-2 V736A	ACAGGACCTGTGCAGCGAGGCCTATCGCTATCAGGTGACGC	GCGTCACCTGATAGCGATAGGCCTCGCTGCACAGGTCCTGT
TMPRSS6-2 V795I	GCCGGCCTAACTACTTCGGCATCTACACCCGCATCACAGGT	ACCTGTGATGCGGGTGTAGATGCCGAAGTAGTTAGGCCGGC
TMPRSS6-2 G603R	TTCGGGGTCGACACATCTGTCGGGGGGCCCTCATCGCTGAC	GTCAGCGATGAGGGCCCCCCGACAGATGTGTCGACCCCGAA

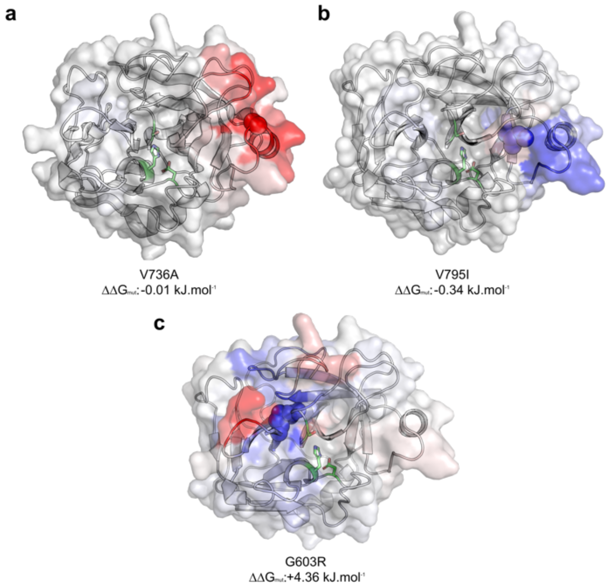

Figure S1. Vibrational entropy predictions of TMPRSS6 variants catalytic domain. TMPRSS6 V736A (**a**), V795I (**b**) and G603R (**c**) vibrational entropy changes were predicted using the crystal structure of the human Matriptase (MTSP1) bound to benzamidine (PDB: 1EAX) as a template for the homology modelling of the TMPRSS6-1/2 WT catalytic domain (located between the residues 577 and 811 of TMPRSS6-1 sequence). TMPRSS6 is shown as gray cartoon and surface. The surface is color-coded according to the difference in vibrational entropy ($\Delta\Delta S_{vib}$) between the variant and the WT enzyme catalytic domain. Red indicates a gain of vibrational entropy while blue indicates a loss of vibrational entropy. The intensity of the color is proportional to the relative vibrational entropy ($\Delta\Delta S_{vib}$) within the protein. (green sticks: catalytic triad, spheres: mutation). Based on the evaluation of thermodynamic parameters, the free energy differences between wild-type and mutants is presented as $\Delta\Delta G_{mut}$ (kJ.mol^{-1}). The G603R mutant is the only variant to present an unfavorable, and possibly destabilizing, modification of its free energy. Numbering refers to the TMPRSS6 isoform 1 catalytic domain sequence (residues 577 to 811).

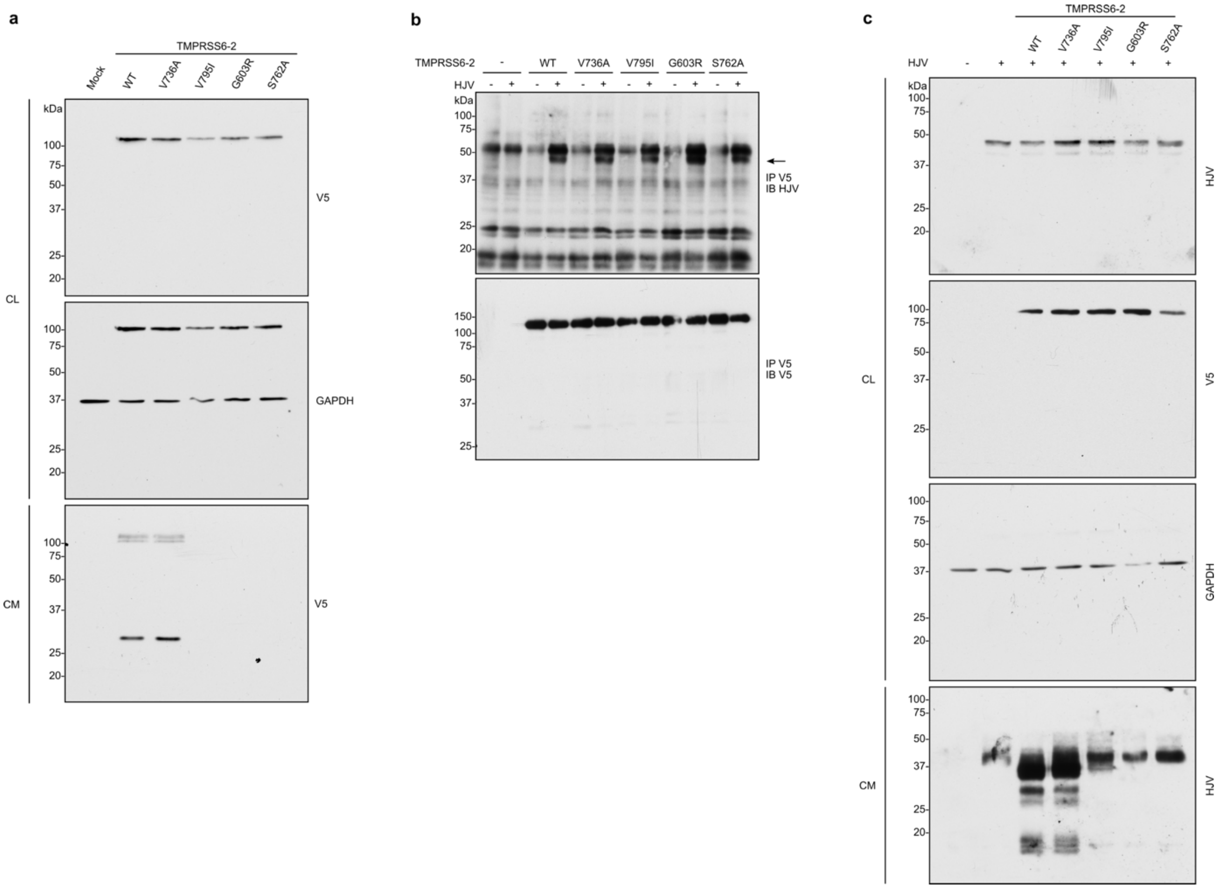

Figure S2. Uncropped blot images from figure 3 (a) and figure 4 (b, c).

References

1 Duchêne D, Colombo E, Désilets A, Boudreault PL, Leduc R, Marsault E *et al.* Analysis of subpocket selectivity and identification of potent selective inhibitors for matriptase and matriptase-2. *J Med Chem* 2014; **57**: 10198–10204.

2 Frappier V, Najmanovich RJ. A coarse-grained elastic network atom contact model and its use in the simulation of protein dynamics and the prediction of the effect of mutations. *PLoS Comput Biol* 2014; **10**: e1003569.

3 Schymkowitz J, Borg J, Stricher F, Nys R, Rousseau F, Serrano L. The FoldX web server: An online force field. *Nucleic Acids Res* 2005; **33**: 382–388.

4 Frappier V, Chartier M, Najmanovich RJ. ENCoM server: exploring protein conformational space and the effect of mutations on protein function and stability. *Nucleic Acids Res* 2015; **43**: W395-400.

5 Frappier V, Chartier M, Najmanovich R. Applications of Normal Mode Analysis Methods in Computational Protein Design. In: *Methods in molecular biology (Clifton, N.J.)*. 2017, pp 203–214.
